# Pharmacological Activation of SIRT3 Modulates the Response of Cancer Cells to Acidic pH

**DOI:** 10.3390/ph17060810

**Published:** 2024-06-20

**Authors:** Michele Aventaggiato, Tania Arcangeli, Enza Vernucci, Federica Barreca, Luigi Sansone, Laura Pellegrini, Elena Pontemezzo, Sergio Valente, Rossella Fioravanti, Matteo Antonio Russo, Antonello Mai, Marco Tafani

**Affiliations:** 1 Department of Experimental Medicine, Sapienza University of Rome, Viale Regina Elena 324, 00161 Rome, Italy; federica.barreca@uniroma1.it (F.B.); 2 Department of Human Sciences and Promotion of the Quality of Life, San Raffaele Roma Open University, Via di Val Cannuta 247, 00166 Rome, Italy; luigi.sansone@sanraffaele.it (L.S.); matteoantoniorusso44@gmail.com (M.A.R.); 3 Laboratory of Cellular and Molecular Pathology, IRCCS San Raffaele Rome, Via di Val Cannuta 247, 00166 Rome, Italy; 4 European Hospital, New Fertility Group, Center for Reproductive Medicine, Via Portuense 700, 00149 Rome, Italy; 5 Department of Drug Chemistry and Technologies, Sapienza University of Rome, Piazzale Aldo Moro 5, 00185 Rome, Italy; sergio.valente@uniroma1.it (S.V.); rossella.fioravanti@uniroma1.it (R.F.); antonello.mai@uniroma1.it (A.M.)

**Keywords:** sirtuins, acidic microenvironment, autophagy, carbonic anhydrase, cancer

## Abstract

Cancer cells modulate their metabolism, creating an acidic microenvironment that, in turn, can favor tumor progression and chemotherapy resistance. Tumor cells adopt strategies to survive a drop in extracellular pH (pHe). In the present manuscript, we investigated the contribution of mitochondrial sirtuin 3 (SIRT3) to the adaptation and survival of cancer cells to a low pHe. SIRT3-overexpressing and silenced breast cancer cells MDA-MB-231 and human embryonic kidney HEK293 cells were grown in buffered and unbuffered media at pH 7.4 and 6.8 for different times. mRNA expression of SIRT3 and CAVB, was measured by RT-PCR. Protein expression of SIRT3, CAVB and autophagy proteins was estimated by western blot. SIRT3-CAVB interaction was determined by immunoprecipitation and proximity ligation assays (PLA). Induction of autophagy was studied by western blot and TEM. SIRT3 overexpression increases the survival of both cell lines. Moreover, we demonstrated that SIRT3 controls intracellular pH (pHi) through the regulation of mitochondrial carbonic anhydrase VB (CAVB). Interestingly, we obtained similar results by using MC2791, a new SIRT3 activator. Our results point to the possibility of modulating SIRT3 to decrease the response and resistance of tumor cells to the acidic microenvironment and ameliorate the effectiveness of anticancer therapy.

## 1. Introduction

Cancer is a multifactorial and complex pathology with a high incidence of death [1]. In the last decades, many protocols have been developed in order to eradicate this disease, but chemotherapies are often not specific and characterized by relevant side effects. Nevertheless, progress in screening, detection, and treatment allows for tight control of this disease, which results in an increased life expectancy [2]. One of the most important features of cancer cells is their ability to modify the extracellular environment by favoring the anaerobic glycolysis pathway even in the presence of oxygen [3]. This ability is known as the “Warburg effect” from the name of Otto Warburg, who first described this attitude of cancer cells [4]. This alternative pathway causes a decrease in the intracellular and extracellular pH, strictly related to the accumulation of lactic acid [5]. Intracellular pH is very important in order to maintain the essential functions of cells, such as membrane permeability, cellular metabolism, proliferation, and enzyme activity. Cancer cells can adapt themselves to different microenvironments in order to survive, especially in low pH conditions [6]. In fact, much evidence have underlined the presence of a low extracellular pH in several models of human cancer, with a shift from 5.9 to 7.2 [7]. The extracellular pH value is an important factor that drives cancer hallmarks of development such as neoangiogenesis, growth, invasiveness, and progression. In fact, low extracellular pH (pHe) causes resistance to therapies and inhibits the activity and efficiency of the immune system [6,8,9,10,11]. Furthermore, cells can control their intracellular pH through the up-regulation of proton pumps and transporters that export hydrogen ions from the cytosol to the extracellular microenvironment [2]. In this context, cancer cells can take advantage of the differential expression of several proteins involved in the homeostasis of the intracellular pH, such as carbonic anhydrase IX and carbonic anhydrase XII for their growth and malignant progression. Carbonic anhydrases are zinc enzymes that consist of five distinct families: alpha, beta, gamma, delta, and epsilon [12] that act as catalysts for the hydration of carbon dioxide to bicarbonate, regulating in this way the acid-base balance in cells [13]. Furthermore, these enzymes are related to CO_2_ and HCO^3−^ transport, bone resorption, ureagenesis, gluconeogenesis, and lipogenesis [14,15]. In particular, the carbonic anhydrases VA (CAVA) and VB (CAVB) are localized in mitochondria [16,17]. In the last few years, CAVB has been shown to have a role in the conversion of CO_2_ produced by the TCA cycle and β-oxidation to HCO^3−^ that finally regulates several metabolic pathways, leading to increased oxidative phosphorylation and reducing reactive oxygen species (ROS) generation [18,19]. Cancer cells, as mentioned above, produce high amounts of H^+^, lactic acid, carbonic acids in order to promote cancer cell metabolism [20,21]. In this context, treatment of cancer in order to eradicate the malignant growth can be based on the inhibition of carbonic anhydrases and proton pumps that cause an increase in pH value [22,23]. Strictly connected with cancer development is the Silent Information Regulator Two (SIRT) family of proteins, called sirtuins, that are NAD+-dependent deacylases conserved from yeast to mammals. In mammals, seven sirtuins (SIRT1–7) have been identified with different subcellular localizations, functions, and targets. SIRT1 and SIRT6 are nuclear; SIRT2 is mainly cytoplasmic; and SIRT3, 4, and 5 are mitochondrial. Finally, SIRT7 is nucleolar [24,25]. SIRT3 controls the levels of global acetylation in mitochondria, taking part in the most important processes that regulate mitochondrial activity, such as ATP balance, the metabolism of nutrients, and oxidative phosphorylation [26,27,28]. For example, SIRT3 can deacetylate and activate acetyl-CoA synthetase 2 (AceCS2), providing a higher quantity of acetyl-CoA to the tricarboxylic acid cycle (TCA) [29,30]. Furthermore, SIRT3 increases the levels of acetyl-CoA through the deacetylation of several enzymes tightly connected with the pyruvate dehydrogenase complex activity [29]. Finally, SIRT3 is involved with the correct activity of the TCA cycle by activating complex II of the electron transport chain and by interacting with complexes I, III, and IV [31,32]. Other important targets of SIRT3 activity are glutamate dehydrogenase (GDH) [33], involved in amino acid oxidation, and the transcriptional factor forkhead box O3 (FOXO3a), which increases the levels of manganese-dependent superoxide dismutase (MnSOD), reducing the levels of ROS [34], involved in cancer transformation. SIRT3 also plays an important role in mitochondrial functions, repair, and respiration and avoids the accumulation of misfolded proteins in mitochondria [35]. Interestingly, SIRT3 has been shown to promote or inhibit cancer development based on the type of cancer and the different signaling pathways [36,37]. In fact, SIRT3 can activate the antioxidant defense system, and the lack of SIRT3 increases ROS signaling pathways, mediating carcinogenesis in several cancer types [38,39]. Furthermore, SIRT3 is involved in the regulation of proapoptotic and antiapoptotic members of the B cell Lymphoma 2 (Bcl-2) family [40]. Several studies have demonstrated a strict interconnection among sirtuins, especially SIRT2, SIRT3, SIRT6, and SIRT7, for their role in protecting cells from several stress stimuli [41,42,43]. In fact, a study conducted by Carnevale et al., demonstrated the presence of an axis between SIRT1 and SIRT3 because the downregulation of SIRT1 causes an increase of SIRT3 in order to protect cells from stress stimuli [44]. The results of these studies have led us to hypothesize an action of SIRT1 in deacetylating and activating ZF5, a transcription inhibitor that, in this way, can bind SP1, causing a decrease in SIRT3 transcription [44]. Finally, another process involved in cancer development is represented by autophagy, a process that can, in turn, suppress or promote carcinogenesis depending on the type of cancer [45,46]. Autophagy, in synergy with the ubiquitin-proteasome system, degrades damaged organelles and misfolded proteins. This process is characterized by autophagosome formation and is regulated by ATG proteins [47]. In several situations, such as nutrient deprivation, hypoxia, pH variation, etc., autophagy is increased in order to guarantee cell survival [48]. Several experiments conducted on cancer cells have shown an activation of autophagy in response to acute and chronic acidosis with increased formation of autophagic vacuoles and a high expression of autophagic markers such as LC3II [48,49]. Furthermore, many studies conducted on cancer cells have demonstrated that low pH increases BNIP3 stability in order to promote cancer survival in acidosis conditions [49,50]. Autophagy activation, in fact, is necessary for cellular survival, while inhibition of autophagy at low pH results in cell death [48,49]. For example, several studies conducted on cancer cells demonstrated that autophagy protects against apoptosis induced by an acidic microenvironment [51]. Taking these aspects into consideration, our hypothesis is that SIRT3 can control cellular response and survival at low pH levels by impinging on CAVB and autophagy. To prove it, we overexpressed and silenced SIRT3 in MDA-MB-231 and HEK293 cell lines, showing increased survival of cells overexpressing SIRT3 at low extracellular pH. After that, we also demonstrated an interaction between SIRT3 and CAVB that is increased at low pH and that it exerts its pro-survival function by activating the autophagic process.

## 2. Results

### 2.1. Viability and Proliferation of MDA-MB-231 and HEK293 Cells under Unbuffered Culture Medium Conditions

**Figure 1:**
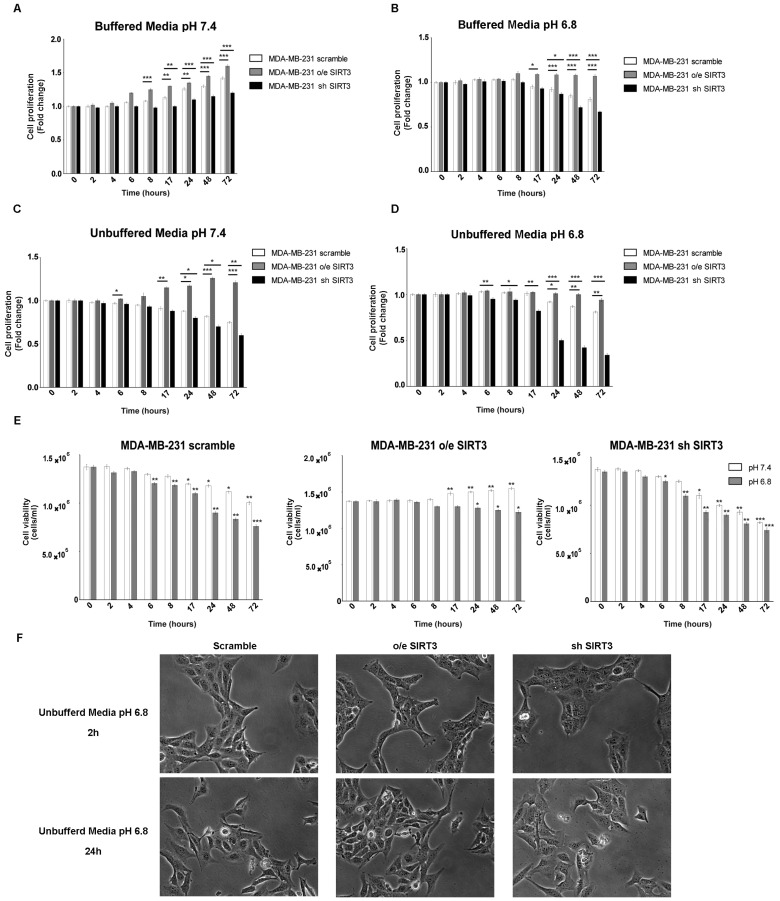
SIRT3 overexpression increases cell survival of MDA-MB-231 in buffered and unbuffered medium. (**A**) MDA-MB-231 proliferation at different time points in buffered media at a pHe of 7.4. (**B**) MDA-MB-231 proliferation at different time points in buffered media at a pHe of 6.8. (**C**) Proliferation rate of MDA-MB-231 clones in unbuffered media at a pHe of 7.4 at different time points. (**D**) Proliferation rate of MDA-MB-231 clones in unbuffered media at a pHe of 6.8 at different time points. (**E**) Cell viability of MDA-MB-231 scramble, o/e SIRT3, and sh SIRT3 after 2 h, 4 h, 6 h, 8 h, 17 h, 24 h, 48 h, and 72 h in unbuffered media at a pHe of 7.4 and 6.8. Data are represented as mean ± SD. * *p* < 0.05, ** *p* < 0.01, and *** *p* < 0.001. (**F**) Images of MDA-MB-231 cells and clones after 2 and 24 h in unbuffered media, pH 6.8 taken at 20× magnification.

It is known that, in tumor cells, the pH gradient from extracellular (acidic) and intracellular (alkaline) compartments are due to their ability to excrete H^+^ protons, acidifying the external microenvironment [52]. The H^+^ secretion depends on the ion exchangers and buffering abilities of cancer cells [53]. SIRT3 “buffer capacity” and the role of this deacetylase in low pH were evaluated by either stably overexpressing or silencing SIRT3 in MDA-MB-231. Protein levels of MDA-MB-231 clones and SIRT3 activity are shown in Figure S1A,B. All three groups of cells were cultured in regular media and unbuffered RPMI at pH 7.4 and 6.8. The proliferation of breast cancer cells was analyzed at different time points (from 2 to 72 h). When cells were cultured in buffered RPMI at pH 7.4, SIRT3-overexpressing cells (o/e SIRT3) showed faster growth kinetics compared to the scrambled and silenced-SIRT3 (sh SIRT3) cells that, in turn, showed the slowest proliferation rate (Figure 1A). Lowering the pH of buffered RPMI to 6.8 reduced cellular growth. However, the same differences in cellular proliferation, described in Figure 1A, among scrambled, overexpressing, and silenced MDA-MB-231 were observed (Figure 1B). Next, cells were grown in unbuffered media as described in [54]. Treatments with unbuffered media induced a reduction in cell proliferation due to a faster pH lowering in cellular media (Figure 1C). In these conditions, differences among clones were more evident. In fact, cells overexpressing SIRT3 showed a significant change in proliferation in unbuffered media at pH 7.4 and 6.8 starting at 24 h of treatment, contrary to scramble, and SIRT3-silenced cells that revealed a decrease in cellular proliferation starting at 6 h after treatment (Figure 1D). Similar results were obtained when assessing cellular vitality. Again, unbuffered media caused a decrease in cell viability that, at pH 7.4, was significant in SIRT3 scrambled and silenced cells from 17 to 72 h (Figure 1E). Unbuffered media at pH 6.8 caused a decrease in cell vitality that started after 8 h and was more marked in SIRT3-silenced cells. On the contrary, SIRT3-overexpressing cells were affected only after 24 h of incubation (Figure 1E). Finally, pictures of cells after 2 and 24 h in unbuffered media show a reduction in cell number in scrambled and SIRT3-silenced cells compared to SIRT3-overexpressing cells (Figure 1F). 

**Figure 2:**
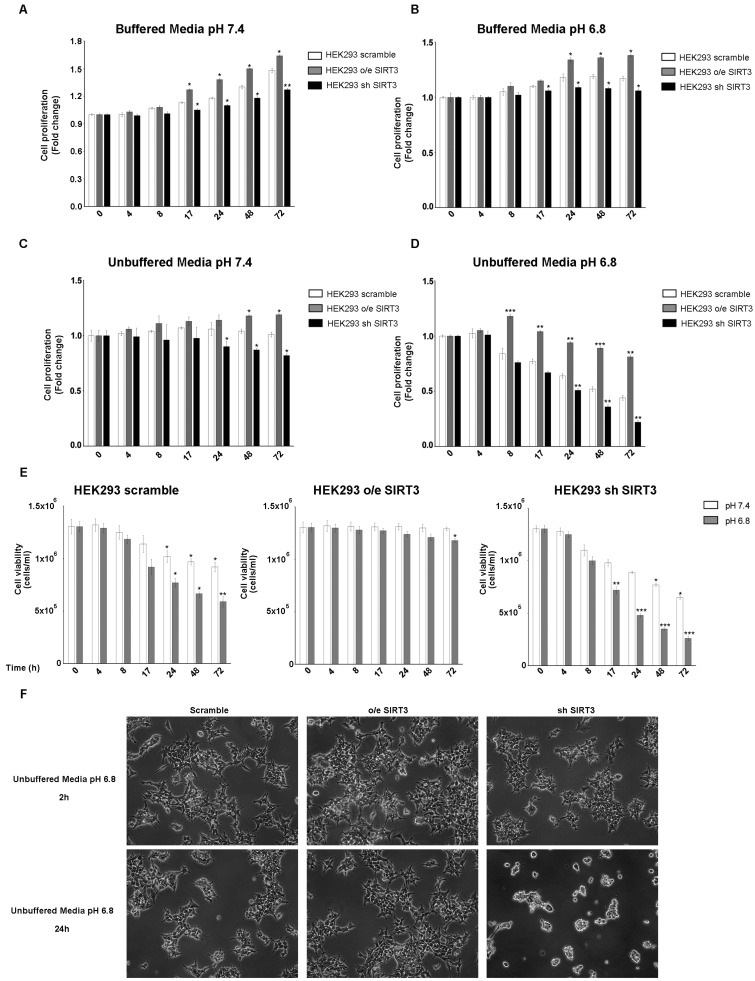
SIRT3 overexpression increases cell survival of HEK293 in buffered and unbuffered medium. (**A**) HEK293 proliferation at different time points in buffered media at a pHe of 7.4. (**B**) HEK293 proliferation at different time points in buffered media at a pHe of 6.8. (**C**) Proliferation rate of HEK293 clones in unbuffered media at a pHe of 7.4 at different time points. (**D**) Proliferation rate of HEK293 clones in unbuffered media at a pHe of 6.8 at different time points. (**E**) Cell viability of HEK293 scramble, o/e SIRT3, and sh SIRT3 after 4 h, 8 h, 17 h, 24 h, 48 h, and 72 h in unbuffered media at a pHe of 7.4 and 6.8. Data are represented as mean ± SD. * *p* < 0.05, ** *p* < 0.01, and *** *p* < 0.001. (**F**) Images of HEK293 cells and clones after 2 and 24 h in unbuffered media, pH 6.8 taken at 10× magnification.

Experiments reported in Figure 1 were repeated, for the same time points, in the HEK293 cells transiently overexpressing or silenced for SIRT3 (Figure S1C,D). Also in this cell line, proliferation was increased after SIRT3 overexpression and decreased after SIRT3 silencing when cells were grown in buffered media at both pH 7.4 and 6.8 (Figure 2A,B). Proliferation was reduced in unbuffered media in HEK293 scrambled and SIRT3 clones (Figure 2C,D). However, scrambled and SIRT3-silenced HEK293 cells showed a marked reduction in proliferation when maintained in unbuffered media at pH 6.8 (Figure 2D). Similarly, also cell viability showed important differences between SIRT3-overexpressing and silenced cells. In fact, SIRT3-silenced cells showed a decrease in cell viability that was not observed in overexpressing cells (Figure 2E). Such marked differences are visible in Figure 2F, which reports pictures of scramble, SIRT3-overexpressing, and SIRT3-silenced cells and shows, after 24 h, a decrease in cell proliferation and vitality in scrambled and SIRT3-silenced cells, respectively (Figure 2F). Taken together, our results suggest a relevant role for SIRT3 in response to extracellular acidosis.

### 2.2. Interplay between SIRT3, CAVB, and Extracellular pH

**Figure 3:**
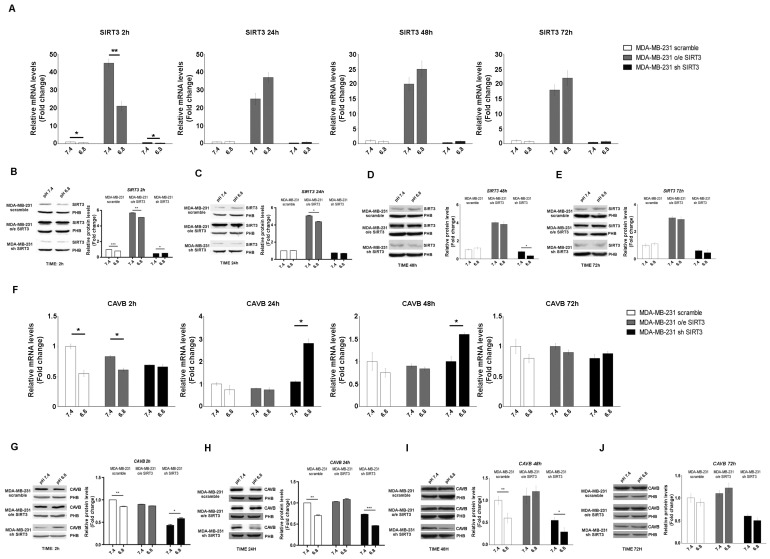
Modulation of SIRT3 and CAVB in MDA-MB-231 during treatment with acidic medium. (**A**) Relative mRNA levels of SIRT3 in MDA-MB-231 clones after different time points of treatments in unbuffered medium at a pHe of 7.4 and 6.8. (**B**) Western blot showing SIRT3 expression in MDA-MB-231 clones after 2 h in unbuffered media at a pHe of 7.4 and 6.8. Prohibitin (PHB) was used as a mitochondrial loading control. Densitometric analysis of SIRT3 protein expression obtained by Western blot after treating MDA-MB-231 clones for 2 h with unbuffered medium at a pHe of 7.4 and 6.8. (**C**) Western blot showing SIRT3 expression in MDA-MB-231 clones after 24 h of treatment with unbuffered media at a pHe of 7.4 and 6.8. Prohibitin was used as a mitochondrial loading control. Densitometric analysis of SIRT3 protein expression obtained by Western blot after treating MDA-MB-231 clones for 24 h with unbuffered medium at a pHe of 7.4 and 6.8. (**D**) Western blot showing SIRT3 expression in MDA-MB-231 clones after 48 h of treatment with unbuffered media at a pHe of 7.4 and 6.8. Prohibitin was used as a mitochondrial loading control. Densitometric analysis of SIRT3 protein expression obtained by Western blot after treating MDA-MB-231 clones for 48 h with unbuffered medium at a pHe of 7.4 and 6.8. (**E**) Western blot showing SIRT3 expression in MDA-MB-231 clones after 72 h of treatment with unbuffered media at a pHe of 7.4 and 6.8. Prohibitin was used as a mitochondrial loading control. Densitometric analysis of SIRT3 protein expression obtained by Western blot after treating MDA-MB-231 clones for 72 h with unbuffered medium at a pHe of 7.4 and 6.8. (**F**) Relative mRNA levels of CAVB in MDA-MB-231 clones after 2, 24, 48, and 72 h of unbuffered medium at a pHe of 7.4 and 6.8. (**G**) Western blot showing CAVB expression in MDA-MB-231 clones after 2 h of treatment with unbuffered media at a pHe of 7.4 and 6.8. Prohibitin (PHB) was used as a mitochondrial loading control. Densitometric analysis of CAVB protein expression obtained by Western blot after treating MDA-MB-231 clones for 2 h with unbuffered medium at a pHe of 7.4 and 6.8. (**H**) Western blot showing CAVB expression in MDA-MB-231 clones after 24 h of treatment with unbuffered media at a pHe of 7.4 and 6.8. Prohibitin was used as a mitochondrial loading control. Densitometric analysis of CAVB protein expression obtained by Western blot after treating MDA-MB-231 clones for 24 h with unbuffered medium at a pHe of 7.4 and 6.8. (**I**) Western blot showing CAVB expression in MDA-MB-231 clones after 48 h of treatment with unbuffered media at a pHe of 7.4 and 6.8. Prohibitin (PHB) was used as a mitochondrial loading control. Densitometric analysis of CAVB protein expression obtained by Western blot after treating MDA-MB-231 clones for 48 h with unbuffered medium at a pHe of 7.4 and 6.8. (**J**) Western blot showing CAVB expression in MDA-MB-231 clones after 72 h of treatment with unbuffered media at a pHe of 7.4 and 6.8. Prohibitin (PHB) was used as mitochondrial loading control. Densitometric analysis of CAVB protein expression obtained by Western blot after treating MDA-MB-231 clones for 72 h with unbuffered medium at a pHe of 7.4 and 6.8. Data are represented as mean ± SD. * *p* < 0.05, ** *p* < 0.01, and *** *p* < 0.001.

In the last few years, it has been demonstrated that carbonic anhydrases (CAs) can generate and maintain the pH gradient in cancer cells [55]. CAs can have different localizations inside the cells [56]. Since our paper focuses on SIRT3, a mitochondrial sirtuin, we investigated the role of CAVB, known to be expressed in the mitochondrial matrix, whose activity is increased by SIRT3 [27]. First, we investigated if the mRNA expression of SIRT3 and CAVB was influenced by pHe and unbuffered conditions. We checked the SIRT3 mRNA level in scrambled, SIRT3-overexpressing, and SIRT3-silenced MDA-MB-231 cells kept in unbuffered medium from 2 to 72 h. After 2 h, SIRT3 expression showed a decrease at a pHe of 6.8 compared to 7.4 both in scrambled and transfected cells (Figure 3A). However, such rapid decrease was not present from 24 to 72 h of treatment (Figure 3A). The same experiment was performed to check SIRT3 protein levels. Again, we observed a decreased in SIRT3 protein after 2 h at a pHe of 6.8 compared to a pHe of 7.4 in the scramble, SIRT3-overexpressing and silenced cells (Figure 3B). Reduced SIRT3 expression a pHe of 6.8 was observed in SIRT3-overexpressing cells after 24 h and in SIRT3-silenced cells after 48 h (Figure 3C,D). Finally, no differences in SIRT3 expression were observed after 72 h or treatment (Figure 3E). Taken together, these results indicate the presence of an adaptation mechanism regulating endogenous SIRT3 expression in the presence of a low pHe. mRNA and protein levels of CAVB were also analyzed. In MDA-MB-231 scrambled and SIRT3-overexpressing cells, the qPCR results showed a significant decrease of CAVB at pHe 6.8 compared to 7.4 (Figure 3F). Interestingly, an increase in CAVB mRNA was observed in SIRT3-silenced cells after 24 h and 48 h of exposure to pHe 6.8 (Figure 3F). This can probably indicate that SIRT3 knockdown cells try to compensate for SIRT3 absence by increasing the expression of CAVB mRNA because of protracted treatment at a pHe below physiological conditions. Analysis of CAVB protein showed a decrease at pHe 6.8, from 2 h to 48 h for the MDA-MB-231 scrambled cells. In the case of SIRT3-silenced cells, the CAVB protein increased at pHe 6.8 after 2 h to then decrease at 24 h and 48 h (Figure 3G–I). Considering the increase in CAVB mRNA in SIRT3-silenced cells, we suppose that there might be some translational control, block, or increased turnover of the CAVB protein that does not allow its accumulation. No changes in CAVB were observed in SIRT3-overexpressing cells (Figure 3G–J). These data suggest that a low pHe, time of exposure to a low pHe, and a lack of SIRT3 can affect CAVB protein expression.

**Figure 4:**
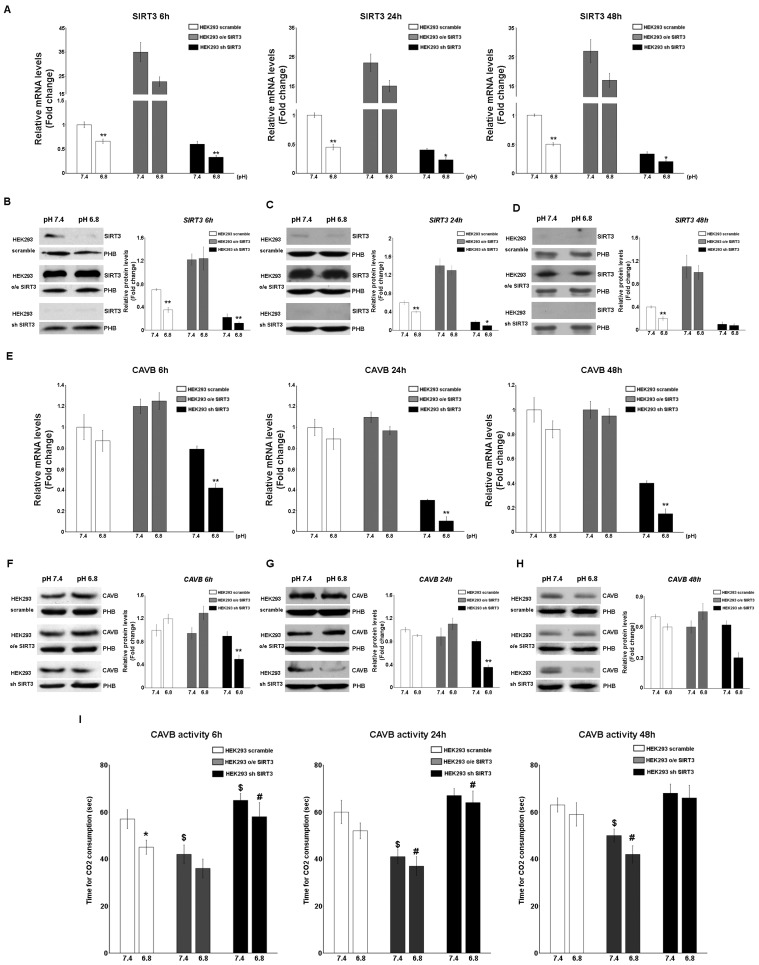
Modulation of SIRT3 and CAVB in HEK293 during treatment with acidic medium. (**A**) Relative mRNA levels of SIRT3 in HEK293 clones after 6, 24, and 48 h of unbuffered medium at a pHe of 7.4 and 6.8. (**B**) Western blot showing SIRT3 expression in HEK293 clones after 6 h of treatment with unbuffered media at a pHe of 7.4 and 6.8. Prohibitin (PHB) was used as a mitochondrial loading control. Densitometric analysis of SIRT3 protein expression obtained by Western blot after treating HEK293 clones for 6 h with unbuffered medium at a pHe of 7.4 and 6.8. (**C**) Western blot showing SIRT3 expression in HEK293 clones after 24 h of treatment with unbuffered media at a pHe of 7.4 and 6.8. Prohibitin (PHB) was used as a mitochondrial loading control. Densitometric analysis of SIRT3 protein expression obtained by Western blot after treating HEK293 clones for 24 h with unbuffered medium at a pHe of 7.4 and 6.8. (**D**) Western blot showing SIRT3 expression in HEK293 clones after 48 h of treatment with unbuffered media at a pHe of 7.4 and 6.8. Prohibitin (PHB) was used as a mitochondrial loading control. Densitometric analysis of SIRT3 protein expression obtained by Western blot after treating HEK293 clones for 48 h with unbuffered medium at a pHe of 7.4 and 6.8. (**E**) Relative mRNA levels of CAVB in HEK293 clones after 6, 24, and 48 h of unbuffered medium at a pHe of 7.4 and 6.8. (**F**) Western blot showing CAVB expression in HEK293 clones after 6 h of treatment with unbuffered media at a pHe of 7.4 and 6.8. Prohibitin (PHB) was used as a mitochondrial loading control. Densitometric analysis of CAVB protein expression obtained by Western blot after treating HEK293 clones for 6 h with unbuffered medium at a pHe of 7.4 and 6.8. (**G**) Western blot showing CAVB expression in HEK293 clones after 24 h of treatment with unbuffered media at a pHe of 7.4 and 6.8. Prohibitin (PHB) was used as a mitochondrial loading control. Densitometric analysis of CAVB protein expression obtained by Western blot after treating HEK293 clones for 24 h with unbuffered medium at a pHe of 7.4 and 6.8. (**H**) Western blot showing CAVB expression in HEK293 clones after 48 h of treatment with unbuffered media at a pHe of 7.4 and 6.8. Prohibitin (PHB) was used as a mitochondrial loading control. Densitometric analysis of CAVB protein expression obtained by Western blot after treating HEK293 clones for 48 h with unbuffered medium at a pHe of 7.4 and 6.8. Student’s *t*-test was used to determine the statistical significance. (**I**) CAVB activity after 6, 24, and 48 h treatment, measured as the time required for the CO_2_ in the Maren buffer to dissolve. Two-way ANOVA with the Bonferroni test was used in (**I**). Data are represented as mean ± SD. * *p* < 0.05, ** *p* < 0.01. *, significantly different from scrambled cells. $, #, significantly different from the other two groups.

SIRT3 and CAVB mRNA and protein expression were also measured in HEK293 cells scrambling, overexpressing, or silenced for SIRT3. In this case, cells were kept in unbuffered media for 6 h, 24, and 48 h in order to avoid excessive cell death, as observed in Figure 2F. Results show that SIRT3 mRNA decreased in scrambled and SIRT3-silenced cells at a pHe of 6.8 compared to a pHe of 7.4 for the times indicated (Figure 4A). Similar results were obtained when measuring the expression of the SIRT3 protein. Again, a decrease in SIRT3 expression at a pHe of 6.8 was observed in both scrambled and SIRT3-silenced cells (Figure 4B–D). As for CAVB, mRNA expression decreased in SIRT3-silenced cells at a pHe of 7.4 and 6.8 (Figure 4E). Similarly, expression of CAVB protein decreased at pH 6.8 in SIRT3-silenced cells (Figure 4F–H). Moreover, such a decrease was accompanied by a decrease of CAVB enzymatic activity that was more pronounced in SIRT3-silenced cells at both pHe 7.4 and 6.8 (Figure 4I). On the contrary, scrambled wt cells showed increased CAVB activity only at a pHe of 6.8 whereas, SIRT3-overexpressing cells showed increased CAVB activity at a pHe of 7.4 and 6.8 (Figure 4I).

### 2.3. SIRT3 Interacts with CAVB

**Figure 5:**
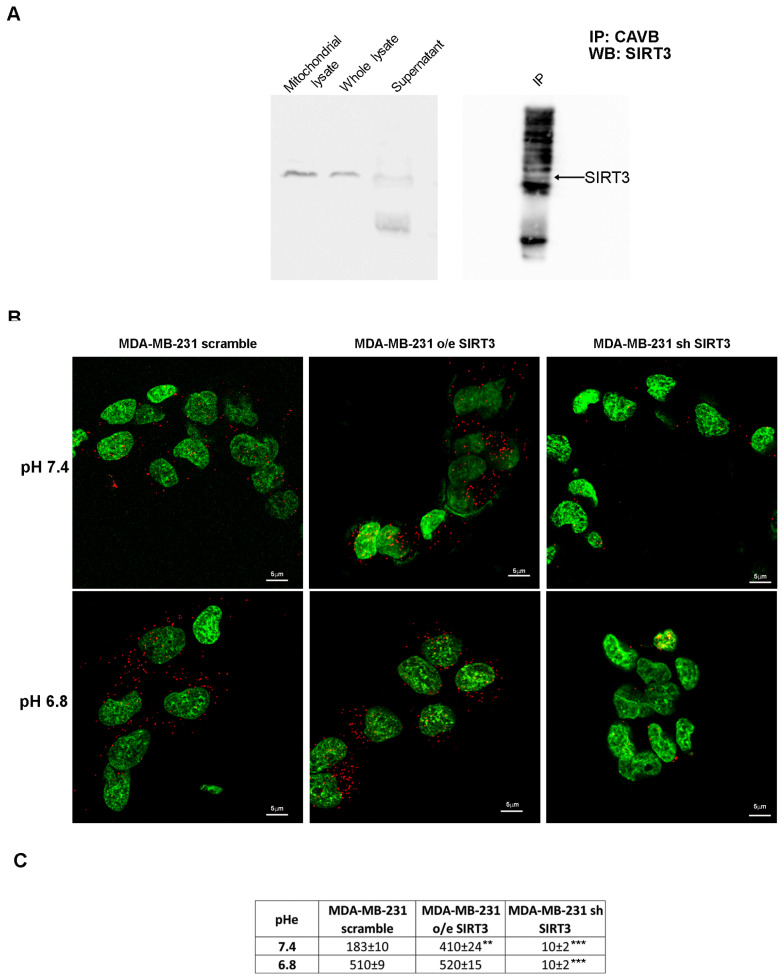
SIRT3 interacts with CAVB. (**A**) Mitochondrial extract from MDA-MB-231 scrambled cells was immunoprecipitated with an anti-CAVB antibody, electrophoresed on an SDS-polyacrylamide gel, and immunoblotted with an anti-SIRT3 antibody. (**B**) Proximity ligation assay of SIRT3 and CAVB; the interactions are shown as red dots, and nuclei are stained with Sytox green. Images were taken at 60×, and the scale bar is indicated in the figure. (**C**) The number of interactions (red dots in the confocal images) for scramble, o/e SIRT3, and sh SIRT3 cells is reported as means ± standard deviations. ** *p* < 0.01, and *** *p* < 0.001. Fifty cells for each group were analyzed using Image J software v1.51 (NIH, Bethesda, MD, USA).

To check for a possible interaction between SIRT3 and CAVB, we performed an immunoprecipitation assay (IP). Our results showed that SIRT3 coimmunoprecipitates with CAVB (Figure 5A). Moreover, in support of our experiment, we compared the SIRT3 expression in the whole lysate and mitochondrial extract before starting the IP and after incubation with the complex beads/CAVB antibody. As shown in Figure 5A, SIRT3 expression decreased, which is as an indication that the protein was seized by CAVB. To confirm our data, we have also performed an in situ proximity ligation assay. This assay revealed that, at pHe 7.4, endogenous SIRT3 and CAVB are close in scrambled cells (Figure 5B). As expected, SIRT3-CAVB interaction was increased in SIRT3-overexpressing cells and decreased in SIRT3 knockdown cells at a pHe of 7.4 (Figure 5B). Interestingly, when the pHe was lowered to 6.8, SIRT3-CAVB puncta increased in both scrambled and overexpressing cells, whereas no difference was observed in knockdown cells (Figure 5B). The quantification of detected interactions is shown in Figure 5C. These data demonstrate that SIRT3 and CAVB coimmunoprecipitate, and the interaction is stronger when cells are in an acidic environment.

### 2.4. Acidic pHe Increases SIRT3 and CAVB Activities but Does Not Affect GDH Activity

**Figure 6:**
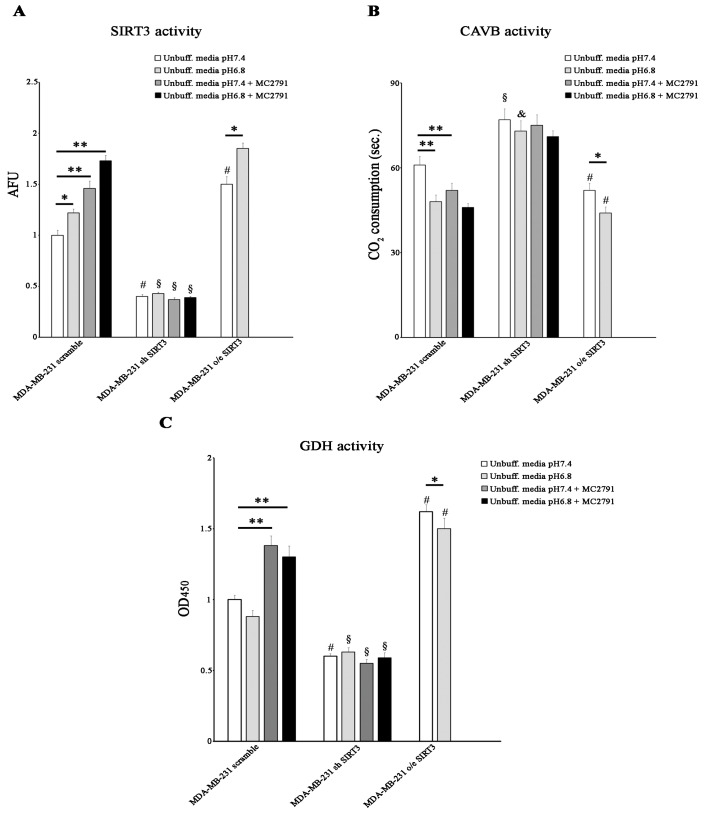
Low pHe increases SIRT3 and CAVB but not GDH activities. (**A**) SIRT3 activity in MDA-MB 231 scramble, o/e SIRT3, and sh SIRT3 cells. (**B**) CAVB activity is measured as the time required for the CO_2_ in the Maren buffer to dissolve. (**C**) GDH activity in MDA-MB 231 scrambled SIRT3-overexpressing and SIRT3-silenced. Data are represented as mean ± SD. The bar charts in (**A**–**C**) were compared by a Two-way ANOVA with the Bonferroni test. Data are represented as mean ± SD. * *p* < 0.05, ** *p* < 0.01. *, significantly different from scrambled cells. #, §, & significantly different from the other two groups.

Since SIRT3 and CAVB are localized both in the mitochondrial matrix and since we have shown, for the first time, that they interact, we hypothesized that SIRT3 could regulate CAVB activity. To better analyze the role of SIRT3, we also treated scrambled and SIRT3-silenced cells with the SIRT activator MC2791 [57]. The effect of MC2791 on MDA-MB-231 scrambled is shown in Supplementary Figure S2, where 50 µM of the compound was able to increase SIRT3, reaching the same effect due to SIRT3 overexpression after 2 h and 4 h of treatment. Moreover, Figure S2 also shows that treatment of scrambled cells with MC2791 increased cell viability at 24, 48, and 72 h treatment similar to what was observed in SIRT3-overexpressing cells. When cells were treated with unbuffered RPMI at pHe 7.4, SIRT3 activity was consistent with protein levels. At pHe 6.8, a significant increase in SIRT3 activity was detected in the scrambled and SIRT3-overexpressing cells. Moreover, we observed that 50 µM of the SIRT3 activator MC2791 increased SIRT3 activity in MDA-MB-231 scrambled cells at both pHe 7.4 and 6.8. These data demonstrate that the acidification of the growth medium induced an increase in the catalytic activity of SIRT3 (Figure 6A). We also measured the CAVB activity in the MDA-MB-231 cell line. CAVB activity was higher in SIRT3-overexpressing cells than in scrambled or silenced cells at both pHe 7.4 and 6.8. Moreover, CAVB activity increased in the scrambled and overexpressing cells in the presence of acidic pH (6.8) (Figure 6B) in accordance with SIRT3 activity (Figure 6A). These results are similar to those obtained in HEK293 cells (Figure 4G). Importantly, treatment with 50 µM MC2791 increased CAVB catalytic activity at pHe 7.4 but not at pHe 6.8 in scrambled cells. On the contrary, we did not observe any change in CAVB catalytic activity in SIRT3-silenced cells when treated with MC2791 (Figure 6B). To check if SIRT3 downstream targets were also upregulated, we investigated the activity of GDH. We determined if acidic pHe could modulate GDH activity in MDA-MB-231 scramble, overexpressing, and silenced cells. As shown in Figure 6C, GDH activity was higher in overexpressing SIRT3 cells at pHe 7.4 and pHe 6.8 compared to scrambled and silenced cells (Figure 6C). However, an increase in GDH activity was observed in the scrambled cells after treatment with MC2791 (Figure 6C). Interestingly, GDH activity was not influenced by pHe 7.4 or 6.8, or by MC2791 in SIRT3-silenced cells (Figure 6C).

### 2.5. SIRT3 Modulates Autophagy in MDA-MB-231 and HEK293 Cells

**Figure 7:**
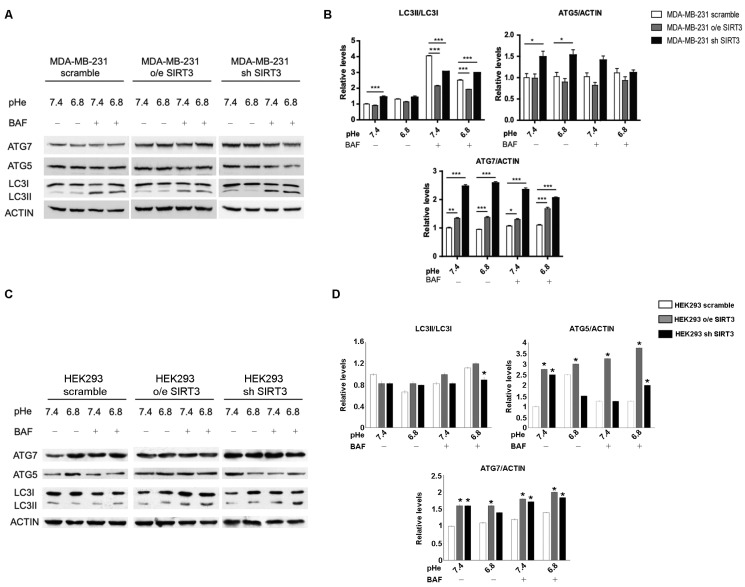
Decreased SIRT3 expression increases autophagy in MDA-MB-231 and HEK293 cells. (**A**) LC3I, LC3II, ATG5, and ATG7 protein expression was analyzed in MDA-MB-231 clones after treatment with unbuffered medium at a pHe of 7.4 and 6.8 for 6 h. During the last two hours, cells were either left untreated or treated with Bafilomicyn A1 (BAF) at 100 nM. Actin was used as a loading control. (**B**) Densitometric analysis of LC3II/LC3I ratio and ATG5 and ATG7 protein expression obtained by Western blot as in (**A**). Data are represented as mean ± SD. * *p*< 0.05, ** *p* < 0.01, and *** *p* < 0.001. (**C**) HEK293 scramble, o/e SIRT3, and sh SIRT3 cells were grown in unbuffered medium at a pHe of 7.4 or 6.8 for 6 h in the presence or absence of Bafilomycin A1 for the last two hours. LC3I, LC3II, ATG5, and ATG7 protein expression was analyzed by Western blot. (**D**) Densitometric analysis of protein expression as in (**C**). Data are represented as mean ± SD. * *p*< 0.05.

Induction of autophagy may represent an adaptive mechanism for cancer cells exposed to an acidic environment [48,58,59] and the ability to maintain a functional autophagic flux in response to pHe changes may vary among different types of cancer [59]. Starting from these considerations, we investigated the autophagic response of MDA-MB-231 scrambled and SIRT3 clones. We measured LC3I, LC3II, ATG5-ATG12, and ATG7 accumulation in the presence or absence of 100 nM Bafilomycin-A1, a vacuolar ATPase inhibitor. LC3II/LC3I ratio, in the absence of Bafilomycin, showed an increase in MDA-MB-231 silenced cells at a pHe of 7.4 (Figure 7A,B). The inhibition of lysosomal acidification and autophagic flux, induced by Bafilomycin-A1, revealed that the LC3II/LC3I ratio was significantly lower in SIRT3-overexpressing cells compared to scrambled and silenced cells at a pHe of 7.4 and 6.8. In addition, the accumulation of LC3II was significantly higher in SIRT3 scrambled cells compared to SIRT3-silenced cells at a pH of 7.4 whereas at pH 6.8, SIRT3-silenced cells increased LC3II accumulation to levels above those of SIRT3 scrambled cells, suggesting an increased flux (Figure 7A,B). Then, we analyzed the ubiquitin-like conjugation systems, considering the expression of ATG7 and ATG5–ATG12 proteins. While no significant changes were observed for ATG5-ATG12, except the increase in SIRT3-silenced cells at both pHs, ATG7 was increased in SIRT3-silenced and overexpressing cells before and after Bafilomycin-A1 treatment (Figure 7A,B). Autophagy induction was also assessed in HEK293 scramble, SIRT3-overexpressing, or silenced cells. In this case, the LC3II/LC3I ratio did not show any significant modulation in unbuffered conditions (Figure 7C,D). However, SIRT3-silenced cells showed increased LC3II accumulation in the presence of Bafilomycin-A1 at a pH of 6.8, suggesting an increase in autophagic flux (Figure 7C,D). Differences were clearer with the ATG7 and ATG5–ATG12 proteins. In fact, we observed an increase in ATG7 in both HEK293 overexpressing and silenced cells compared to scrambled cells in the presence or absence of Bafilomycin at pH 7.4 and 6.8 (Figure 7C,D). ATG5–ATG12 levels increased only in SIRT3-overexpressing cells compared to scrambled and silenced ones (Figure 7C,D). In the presence of Bafilomycin, such a difference was maintained, with SIRT3-silenced cells accumulating more ATG5–ATG12 compared to scrambled cells (Figure 7C,D).

**Figure 8:**
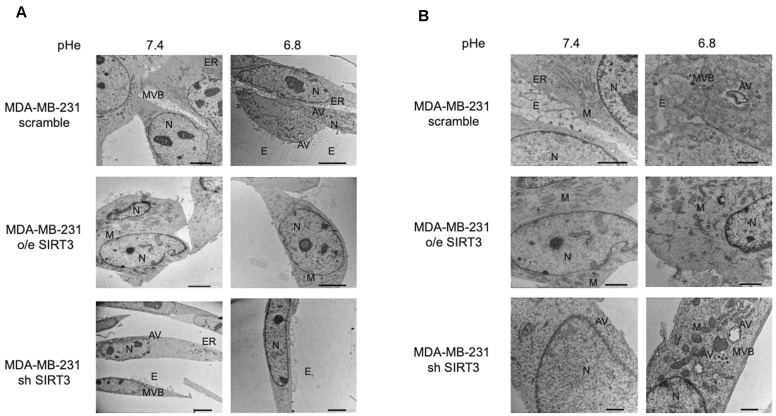
Morphology and autophagosome formation in MDA-MB-231 scramble, o/e SIRT3, and sh SIRT3 cells. (**A**) MDA-MB-231 scramble, o/e SIRT3 and sh SIRT3 cells were grown in unbuffered medium at pHe 7.4 or 6.8 for 8 h. Afterward, cells were analyzed by transmission electron microscope (TEM). (**B**) High magnification images of MDA-MB-231 clones by TEM. N = nucleus, M = mitochondria, ER = Endoplasmic Reticulum, E = Exosome, MVB = Multi vesicular body, AV = autophagic vacuole. Scale bar = 10 µm.

Taken together, these results suggest that presence or absence of SIRT3, more than the acidic microenvironment, modulates the autophagic process in MDA-MB-231 and, partially, in HEK293 cells. Finally, to better investigate this aspect, we studied autophagy using transmission electron microscopy (TEM), which remains one of the most widely used and sensitive techniques to monitor autophagy. MDA-MB-231 SIRT3-silenced cells, when cultured with unbuffered medium, show an elongated morphology, cytoplasm with few organelles, and baggy mitochondria compared to scrambled and SIRT3-overexpressing cells (Figure 8A). Moreover, consistent with previous western blot results, we can see that scrambled and SIRT3-silenced cells show more autophagic vacuoles compared to SIRT3-overexpressing cells (Figure 8B).

## 3. Discussion

Altered metabolism represents one of the cancer hallmarks [60], and in some tumors, it can identify a pre-cancerous status [61]. Metabolic rewiring in cancer cells towards a glycolytic phenotype [62] culminates in massive production of lactic acid that, along with reduced blood perfusion, contributes to creating an acidic tumor microenvironment [63]. Tumor acidosis, besides supporting the pathogenesis and the tumor progression, can act as a mechanism to regulate therapy effectiveness and chemotherapy resistance [64,65]. In this scenario, it is beneficial to identify the targets that can confer an advantage in the acidic microenvironment of cancer cells. In our study, we highlighted the role of SIRT3 in protecting MDA-MB-231 and HEK293 cells from acidic stress. The breast cancer cell line MDA-MB-231 and the immortalized kidney cell line HEK293 represent two completely different tissue origins. In fact, such a difference is visible in the kinetics of cell death as well as mRNA and protein expression following incubation in unbuffered or acidic media (Figure 1, Figure 2, Figure 3 and Figure 4). However, this is exactly the reason why we chose these two cell lines: to prove that, notwithstanding such intrinsic differences, they respond to a drop in extracellular pH by activating a common pathway that has as major players SIRT3 and CAVB. This suggests that the SIRT3-CAVB axis is conserved among cell lines, although probably more robust in cancer cells that have evolved in an acidic microenvironment. In particular, we have observed that SIRT3-overexpressing cells were able to contrast the lowering of extracellular pH longer than scrambled and silenced cells (Figure 1). We speculate that, through the interaction between SIRT3 and CAVB increased by a low pHe, MDA-MB-231 and HEK293 cells can better survive hostile conditions. It is known that cancer cells are characterized by an alkaline pHi that differs from the more acidic pHe [52]. This equilibrium should be tightly regulated to avoid disrupting biological and biochemical processes essential for cancer to survive and metastasize [66]. It must be kept in mind that the interaction between SIRT3 and CAVB is only one of the many pathways influenced by these sirtuins. In fact, the role of SIRT3 in cancer is somewhat controversial since this protein can work as both an oncogene and an oncosuppressor, depending on the cancer type and history. Such a controversial aspect has been the subject of a large number of papers and reviews [39,67,68,69,70]. Overexpression of SIRT3 has been observed in different types of carcinomas, where it correlates with malignancy and a worse prognosis [71]. On the other hand, downregulation of SIRT3 has also been reported to increase tumor growth and survival [72]. However, this is not totally surprising considering the large number of SIRT3 substrates that, once deacetylated, can act as tumor promoters or suppressors, with the final outcome probably related to which pathway is exploited by the tumor cells. In addition, the timing of cancer development in which SIRT3 is analyzed is also important. In fact, SIRT3 is known to decrease ROS accumulation by deacetylating and activating MnSOD [72] as well as HIF-1α [73], an effect that may initially contrast cellular transformation. However, if cancer cells produce ROS through other pathways or in response to external stresses, then SIRT3 accumulation can be exploited by cancer cells to maintain ROS at a non-toxic level. In conclusion, the level of SIRT3 measured in a given cancer type may depend not only on the type of cancer but also, in the same cancer, on the moment during tumor history at which the analysis is performed.

In a previous paper from our laboratory, we have already demonstrated that SIRT3-overexpressing cells can contrast pHi drops, caused by hypoxia, increasing CAVB activation [27]. In the current study, we observed that MDA-MB-231 cells, when exposed to an acidic pHe, adopt the same mechanism, and, probably, the increased activation of CAVB and production of HCO^3−^ consequently, can represent one of the numerous mechanisms that allowed breast cancer cells to survive while maintaining a beneficial acid-base balance between intracellular and extracellular spaces. It is known that passive and active transports cooperate for pH homeostasis [74,75]. Carbonic anhydrases such as CAIX [76], Na^+^/HCO^3−^ co-transporters [77], the monocarboxylate transporters [78], and the Na^+^-driven Cl^−^/HCO^3−^ exchanger [79] are some of the plasma membrane molecules that change their expression and/or activity to facilitate H^+^ efflux and safeguard the alkaline intercellular pH. Among all the CAs, mitochondrial carbonic anhydrases are still poorly studied, and there is not much information available to understand their importance in cancer. In 2011, a study on PBMC (Peripheral Blood Mononuclear Cells) identified that the deregulation of CAVB, along with the other seven genes, can be used to discriminate between pancreatic cancer patients and healthy people [80], highlighting, in this way, a role of CAVB in cancer recognition.

In our hands, HEK293 cells survived in an acidic, unbuffered medium for up to 24 h. Importantly, SIRT3 overexpression increased cell survival and proliferation. On the contrary, SIRT3 silencing reduced cell survival and proliferation (Figure 2). Similar to MDA-MB-231 cells, HEK293 overexpressing SIRT3 increased CAVB activity (Figure 4). In fact, HEK293 cells can survive in acidic conditions, a situation that has been exploited to increase transfection efficiency [81]. Moreover, HEK293 cells can form tumors in nude mice per se or when transfected with empty vectors [82].

Given the importance of SIRT3 in the control of many intracellular metabolic and non-metabolic pathways, a great deal of effort has been put into the discovery and production of sirtuins modulators [83,84]. In fact, modulation of sirtuins may be important not only in cancer but also in metabolic diseases such as obesity, diabetes, etc., as well as in aging. The growing list of sirtuin modulators includes both activators such as resveratrol and its derivatives resVida and SRT501, or the activator SRT1720, among others, as well as inhibitors such as sirtinol, splitomicin, suramin, etc. [83,84]. It must be noted that almost all of the modulators have SIRT1 as a main target [84]. Some of the inhibitors working on SIRT1 have also been shown to inhibit SIRT3 [84], with 3-TYP being used as a more specific SIRT3 inhibitor [85]. In our study, we have also evaluated the effect of MC2791, a new and validated SIRT3 activator, on CAVB at an acidic pH [57]. We have noticed that MC2791 acting on SIRT3 increases its activity, mainly when it is used in combination with an acidic unbuffered medium (Figure 6). The use of sirtuin modulators represents a therapeutic alternative to drug-antibody conjugates that can be inactivated by host-produced antibodies or CAR-T therapies characterized by side effects such as cytokine production and secondary tumor formation [86,87].

Many studies describe autophagy as an adaptive mechanism that cancer cells can adopt when exposed to acidic pH [48,58,59]. To check the interaction between autophagy, low pH, and SIRT3 alteration, we evaluated the expression of LC3I, LC3II, ATG5 and ATG7 (Figure 7). In our study, we observed an increase in basal levels of autophagic markers in SIRT3-silenced MDA-MB-231 and HEK293 cells. In fact, SIRT3-silenced cells show an accelerated autophagic flux, maybe because they cannot restore physiological pHi due to the lack of the SIRT3-CAVB system or because SIRT3 is involved in autophagy control and its downregulation can interfere with the correct modulation of this process. When we analyzed the LC3II/LC3I ratio at different pHe values, we observed higher levels in scrambled and SIRT3-overexpressing cells at a pHe of 6.8 compared to a pHe of 7.4, a result that is in accordance with the increased SIRT3 activity at a lower pHe (Figure 6), which can also explain why there are no changes in SIRT3-silenced cells. Consistent with an activation of autophagy and with western blot analysis, TEM analysis performed in MDA-MB-231 cells after 8 h treatment at low pH revealed that wt and SIRT3-silenced cells show more autophagic vacuoles compared to SIRT3-overexpressing cells (Figure 8).

## 4. Materials and Methods

### 4.1. Cell Cultures and Reagents

The MDA-MB-231 Human Breast Carcinoma cell line and the HEK293 human embryonic kidney cell line were purchased from LGC Standards (Milan, Italy) and maintained in 10-cm^2^ polystyrene dishes (Corning Costar Corp., Oneonta, NY, USA) with RPMI 1640 medium (Mediatech, Inc., Herndon, VA, USA), supplemented with 2 mM L-Glutamine, 100 units/mL penicillin, 0.1 mg/mL streptomycin, and 10% heat-inactivated fetal bovine serum. Cells were maintained at 37 °C in a humidified atmosphere of 5% CO_2_ and 95% air. Bafilomycin A1 (B1793, Merck, St. Louis, MO, USA) was dissolved in DMSO and added to a final concentration of 100 nM for the times indicated.

### 4.2. Generation of Transient and Stable SIRT3 Transfectants

MDA-MB-231 cells were stably transfected with a pcDNA3.1 expression vector encoding for human SIRT3-Flag (Addgene Inc., Cambridge, MA, USA). Stable clones were generated by delivering plasmid DNA constructs into cells using TurboFectin 8.0 (Origene Technologies, Rockville, MD, USA). Cells were seeded on a 24-well plate. The following day, the cells were transfected according to the manufacturer’s guidelines. Briefly, TurboFectin reagents were first mixed with serum-free RPMI at room temperature for 5 min. Subsequently, plasmid DNA was added to the TurboFectin-containing media and incubated at room temperature for 30 min. After that, the mixtures were added to the cells. The selection of stable clones was started 24 h later with the addition of 500 μg/mL of Geneticin (Merck, St. Louis, MO, USA). Once selected, clones were isolated and grown individually. Transient transfection of HEK293 cells was obtained with the same procedure and reagents described above. Experiments on HEK293 cells were performed within the third day after transfection. SIRT3 expression was confirmed by western blotting analysis in both cell lines.

### 4.3. Lentiviral Transduction

Mission TRC short hairpin RNA (shRNA) lentiviral transduction particles expressing shRNA targeting SIRT3 and lentiviral negative control particles were purchased from Merck. Stably transduced clones were generated according to the manufacturer’s instructions. Cells were seeded on a 24-well plate, and the following day, cells were infected. After 24 h medium was changed with fresh RPMI. The selection of stable clones was started 24 h later with the addition of 3 mg/mL of puromycin. Transient SIRT3 silencing of HEK293 cells was obtained with the same procedure and reagents described above. Experiments on HEK293 cells were performed within the third day after transfection. SIRT3 silencing was confirmed by western blotting analysis in both cell lines.

### 4.4. Development of an Acidic Microenvironment

MDA-MB-231 and HEK293 wt and transfected cells were maintained in buffered medium (pH 7.4), buffered acidic medium (pH 6.8 or 6.6), or unbuffered medium (w/o sodium bicarbonate, initial pH 7.4) as specified through the text. The pH was monitored at the beginning and end of each experiment using a pH meter (Hanna Instruments, Milan, Italy).

### 4.5. Cellular Viability and Proliferation Assay

Cell viability of MDA-MB-231 and HEK293 was measured by Trypan Blue (TB) (0.4% Invitrogen, Waltham, MA, USA) staining. The same number of scrambled, SIRT3-overexpressing, and SIRT3-silenced cells were plated in 10-cm^2^ polystyrene dishes (Corning Costar Corp., Oneonta, NY, USA) in R0883 complete medium. The following day, the cells were exposed to R6504 at pH 7.4 and 6.8 for the indicated time. Cells cultured in RPMI R0883 at the same experimental time points were used as controls. After treatment, cells were washed with PBS and collected. Cells were then transferred to a Falcon^®^ tube. Ten microliters of cell suspension were mixed with ten microliters of TB, and then viable cells were counted. Cell proliferation was measured using the CellTiter 96^®^AQueous One Solution Cell Proliferation Assay (MTS) (Promega, Milan, Italy) following the manufacturer’s instructions. Briefly, the assay was performed by adding a small amount of the CellTiter 96^®^AQueous One Solution Reagent directly to culture wells, incubating for 1 h and then recording the absorbance at 490 nm with the GloMax^®^-Multi Detection System (Promega). The cell viability and proliferation experiments were repeated three times.

### 4.6. Protein Extraction, Western Blotting Assay, and Antibodies

For whole cell lysate, cells (1 × 10^6^) were pelleted at 1200 rpm (5 min at 4 °C) and lysed with Lysis buffer (50 mM TrisHCl pH 7.4, 5 mM EDTA, 250 mM NaCl, 0.1% Triton, 50 mM NaF, 0.1 mM sodium orthovanadate, 1 mM phenylmethylsulfonyl-fluoride (Merck, St. Louis, MO, USA), and 1× protease inhibitor mixture (P8340, Merck, St. Louis, MO, USA). After 30 min on ice, the lysates were clarified by centrifugation (10 min at 4 °C) and the supernatant collected. Protein concentration was determined by the Bradford assay (Bio-Rad, Hercules, CA, USA). Fifty micrograms of protein for each sample were electrophoresed on SDS-polyacrylamide gels. Gels were then blotted onto PVDF membranes. After blocking with 5% milk, membranes were incubated with the primary antibody overnight. Finally, protein expression was analyzed by staining with the appropriate secondary horseradish peroxidase-labeled antibody for 1 h followed by enhanced chemiluminescence detection. The following primary antibodies were used: SIRT3 (1:1000, anti-rabbit; Cell Signaling, Danvers, MA, USA), CAVB (1:500, anti-mouse; Santa Cruz, Dallas, TX, USA), LC3B (1:1000, antirabbit; MBL International, Woburn, MA, USA), ATG5/12 (1:1000, anti-rabbit; Cell Signaling Technology, Danvers, MA, USA), ATG7 (1:500, anti-rabbit; Novus Biologicals, Centennial, CO, USA), β-Actin (1:10,000, anti-mouse; Merck), and Prohibitin (1:1000, anti-mouse; Novus Biological, Centennial, CO, USA). Each Western Blot was repeated three times.

### 4.7. Isolation of Cytosolic and Mitochondrial Fractions

Cells were plated in 10-cm^2^ dishes. Following treatment, the cells were scraped off the plate and centrifuged at 1200 rpm for 10 min at 4 °C. Cell pellets were resuspended in 1 mL of 20 mM HEPES-KOH, pH 7.5, 10 mM KCl, 1.5 mM MgCl_2_, 1 mM EDTA, 1 mM EGTA, 1 mM phenylmethyl-sulfonyl fluoride, 1× protease inhibitor mixture, and 250 mM sucrose (all reagents were from Merc St. Louis, MO, USA). The cells were broken open with six passages through a 26-gauge needle applied to a 1 mL syringe. The homogenate was centrifuged at 1000× *g* for 5 min at 4 °C to harvest the nuclei fraction. The supernatant was transferred to a high-speed centrifuge tube and centrifuged at 10,000× *g* for 25 min at 4 °C. The resulting supernatant (cytosolic fraction) was concentrated through a Microcon YM-10 Centrifugal Filter Device (Millipore, Bedford, MA, USA), whereas the pellet (mitochondrial fraction) was lysed in RIPA Buffer (50 mM TrisHCl, pH 7.4, 150 mM NaCl, 1% Triton X-100, 1 mM EDTA, 0.1% Nonidet P-40, 1 mM phenylmethylsulfonyl fluoride and 1X protease inhibitor mixture (Merck, St. Louis, MO, USA). The protein concentration of the fraction was determined by a Bradford assay (Bio-Rad, Hercules, CA, USA).

### 4.8. RT-PCR

Total RNA was extracted from cells using TRIzol (Invitrogen, Carlsbad, CA, USA). The quality of the RNA was analyzed using an Agilent 2100 Bioanalyzer (Agilent, Santa Clara, CA, USA) and a Nanodrop1000 (ThermoFisher, Waltham, MA, USA). Total RNA (1 μg) was retro-transcribed into complementary DNA according to the manufacturer’s instructions (Invitrogen SuperScript III, Carlsbad, CA, USA). Real Time RT-PCR amplification was carried out in the 7900 Real-time PCR system (Applied Biosystem, Foster City, CA, USA) using Real Time SyBrGreen RqPCR Superscript (Invitrogen, Milan, Italy). 18S expression was used as an endogenous control and for normalization. All reactions were carried out in triplicate. Primers used are summarized in Table 1.

### 4.9. Immunoprecipitation Assay

Proteins were extracted, and the concentration was measured as described above. 2 µg of Mouse Monoclonal Antibody CAVB (IgG, G-1 Santa Cruz, Biotechnology, Dallas, TX, USA) and 20 µL of Protein A/G PLUS-Agarose (beads, Santa Cruz Biotechnology, sc-2003 Dallas, TX, USA) were brought to a final volume of 1 mL with complete Lysis buffer and kept on a rotator overnight at 4 °C. Then, lysate was first precleared with 980 µg of appropriate control IgG and 60 µL of Protein A/G PLUS-Agarose (Santa Cruz Biotechnology, sc-2003 Dallas, TX, USA), brought to a final volume of 1 mL with Lysis buffer, and kept on a rotator for 4 h at 4 °C. Precleared lysates were then added to the beads linked to the CAVB antibody and kept on a rotator overnight at 4 °C. Following incubation, lysates were centrifuged at 6000 rpm for 5 min at 4 °C. The pellet was washed 4 times with Lysis buffer and finally resuspended in 20 µL of 2× Laemmli solution (4% SDS, 20% Glicerol, 0.004% bromophenol blue, 0.125 M TrisHCl (pH 6.8), and 10% β-mercaptoethanol (Merck)). After denaturing for 5 min at 95 °C, the samples were electrophoresed on a 12% SDS-polyacrylamide gel.

### 4.10. Proximity Ligation Assay

To allow further and more detailed studies of SIRT3-CAVB interactions, we have utilized Duolink (Duolink, using PLA Technology, Merck) based on the in situ proximity ligation assay (PLA) technique. Proximity and ligation assays can be used to detect, quantify, and determine the cell localization of protein interactions and their modifications in a single experiment. Briefly, cells were counted and plated on coverslide glass in a 6-well plate in unbuffered complete medium and incubated overnight at 37 °C and 5% CO_2_. Afterwards, cells were incubated with media at different pHs (7.4 and 6.8) for 2 h. After that, cells were fixed (paraformaldehyde 4%), permeabilized (0.5% Triton), and blocked (BSA 1%) (Merck). PLA was performed following the manufacturer’s protocol. The primary antibodies used are Rabbit Monoclonal Antibody SIRT3 (1:100 Cell Signaling) and Mouse Monoclonal Antibody CAVB (1:50 Santa Cruz, Biotechnology, Dallas, TX, USA), while the secondary antibodies are conjugated with oligo-nucleotides. Anti-Rabbit MINUS (Duolink In Situ PLA Probe Affinity purified Donkey anti-Rabbit IgG) to detect SIRT3 and Anti-Mouse PLUS (Duolink In Situ PLA Probe Affinity purified Donkey anti-Mouse IgG) to detect CAVB. Nuclei were stained with Sytogreen (LifeTechnologies, Carlsbad, CA, USA) at 40 nM for 10 min at room temperature. Coverslips were mounted on antifade mountant (ProLong Diamond Antifade Mountant, Life Technologies, Carlsbad, CA, USA). Fluorescence intensity was visualized with a LSM 510 confocal microscope (Zeiss, Oberkochen, Germany). Images were analyzed using ImageJ to calculate the density of PLA puncta. The PLA assay was repeated three times.

### 4.11. SIRT3 Activator

**Figure 9:**
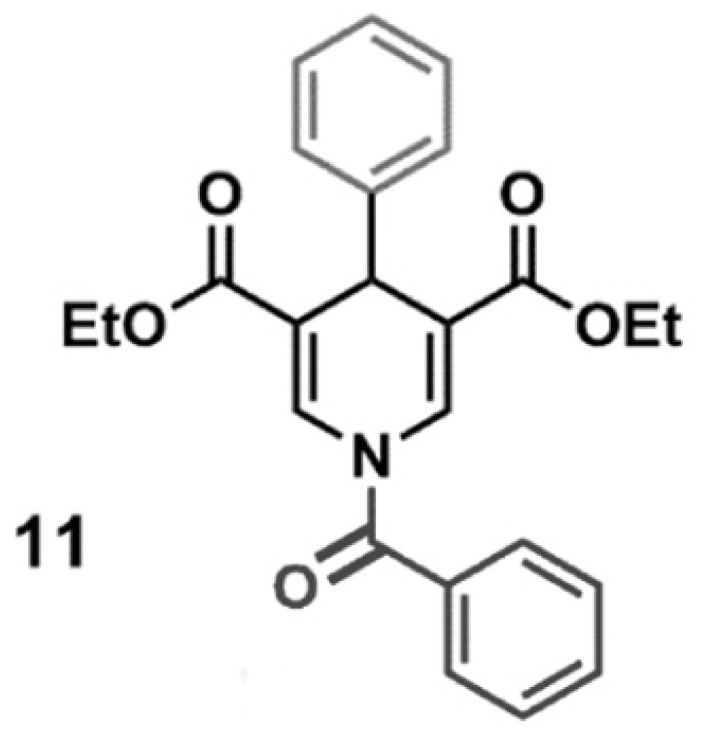
Chemical structure of SIRT3 activator MC2791.

SIRT3 activator (MC2791) is a small molecule able to induce SIRT3 activation. The chemical structure of MC2791 is reported in Figure 9. The synthesis and validation of MC2791 were previously described by us as compound 11 in ref. [57]. In the experiments here reported, MC2791 was dissolved in DMSO, and it was verified that the concentration of DMSO used did not cause cellular toxicity. In all the experiments, 50 µM of MC2791 activator was added to the cells.

### 4.12. SIRT3 Deacetylase Activity Assay

SIRT3 deacetylase activity was determined using the SIRT3 Fluorimetric Activity Assay/Drug Discovery Kit (ENZO Life Technologies, Farmingdale, NY, USA) and following the manufacturer’s instructions. Briefly, MDA-MB-231 scramble, o/e SIRT3, and sh SIRT3 cells were counted and plated on 10-cm^2^ polystyrene dishes in complete medium and incubated overnight at 37 °C and 5% CO_2_. Then the medium was substituted with R6504 at pH 7.4 or 6.8 for 2 h. Afterward, mitochondrial extracts of scramble, o/e SIRT3, and sh SIRT3 cells (40 μg) were incubated with the “Fluor de Lys substrate” buffer at 37 °C for 40 min, followed by incubation with “fluor de lys developer” at 37 °C for 10 min. After excitation at 360 nm, emitted light was detected at 460 nm using an Infinite 200 microplate fluorometer (Tecan, Mannedorf, Switzerland). The fluorescence intensity of the assay buffer was subtracted from each experimental sample. Each SIRT3 assay was repeated three times.

### 4.13. CAVB Activity Measurements

CAVB catalytic activity was determined using the slightly modified end-point titration method of Maren [88]. Briefly, mitochondrial extracts (40 μg) from MDA-MB-231 scramble, o/e SIRT3 and sh SIRT3 cells were resuspended in 500 µL of ice-cold assay buffer (20 mM imidazole, 5 mM TRIS Base, 0.2 mM p(4)-nitrophenol (Merck)). The cuvette containing the sample and assay buffer was placed in a Lambda 35 UV/VIS (Perkin Elmer Instruments, Waltham, MA, USA) spectrophotometer, and 500 µL of ice-cold CO_2_-saturated H_2_O were added to the cuvette. The exact time in seconds for the yellow color disappearance was counted. In the control experiments without cells or extracts, the color disappeared in about 70 s. Each measurement was repeated three times.

### 4.14. Glutamate Dehydrogenase Activity Assay

The enzymatic activity of GDH was determined using the GDH activity assay kit (Merck, MAK099) according to the manufacturer’s protocol. Briefly, cells (1 × 10^6^) were homogenized in 40 µL of ice-cold GDH Assay Buffer, incubated for 10 min on ice, and centrifuged at 13,000× *g* for 5 min. MDA-MB-231 scramble, o/e SIRT3, and sh SIRT3 cells were incubated with 100 µL of Master Reaction Mix and incubated at 37 °C. Following 3 min of incubation, measurements were taken after 5, 15, 30, and 60 min using an Infinite 200 microplate fluorometer (Tecan, Mannedorf, Switzerland). Each measurement was repeated three times.

### 4.15. Transmission Electron Microscopy (TEM)

Cells were cultured at pH 7.4 or 6.8 for 8 h and then processed for Transmission Electron Microscopy (TEM). Briefly, treated cells were fixed with 2.5% glutaraldehyde (Electron Microscopy Sciences, Hatfield, PA, USA) in 0.1 M phosphate buffer overnight at 4 °C and then scraped from the dishes, washed, and subsequently fixed in 1% osmium tetroxide (Electron Microscopy Sciences, Hatfield, PA, USA). After dehydration in a graded series of acetone, the cells were embedded in epoxy resin (Epon-812) (Electron Microscopy Sciences, Hatfield, PA, USA). After polymerization of the resin at 60 °C for two days, thin sections of the cells (80 nm) were cut using an ultramicrotome and stained with lead hydroxide. The sections were then examined and photographed using a transmission electron microscope (TEM) JEM 1400 Plus (JEOL, Akishima, Tokyo, Japan).

### 4.16. Statistical Analysis

The results are expressed as mean ± S.D. and 95% confidence intervals (95% CI). A Student’s *t*-test was used to determine any significant differences between two groups (before and after treatment). The significance was set as * *p* < 0.05, ** *p* <0.01, and *** *p* < 0.001. A Two-way ANOVA with the Bonferroni test was used to determine the effects between the three groups. GraphPad Prism5 and the SPSS statistical software package (SPSS Inc., Version 13.0.1 for Windows, Chicago, IL, USA) were used for all statistical calculations.

## 5. Conclusions

Overall, our paper highlights a new SIRT3 role in regulating the response and survival of tumor cells to acidic pHe by modulating the activity of mitochondrial CAVB. At a lower pHe, SIRT3 interacts with and activates CAVB, which converts the CO_2_ produced by the TCA cycle and β-oxidation to HCO^3−^, concurring to maintain the tumor pH gradient. Moreover, SIRT3 activity is exploited by tumor cells to survive in an acidic microenvironment through the modulation of the autophagic process. Although further investigations are needed, our study lays the groundwork to consider SIRT3 as a new target to counteract the advantages for tumor proliferation deriving from an acidic microenvironment and, consequently, increase therapy sensitivity, therefore suggesting the possible use of SIRT3 modulators as a novel cancer therapeutic strategy.

## Figures and Tables

**Table 1:** Primers used for RT-PCR.

Accession Nb.	Primer	Length	Position	Tm	%GC	Sequence
NM_012239.5 (SIRT3)	Left	20	940–959	59	45	cttgctgcatgtggttgatt
	Right	18	1011–1028	60	56	cggtcaagctggcaaaag
NM_007220 (CAVB)	Left	21	716–736	60	43	tgccgtcaattaagcataagg
	Right	19	777–795	60	58	atctgggcaggtaggcatc

## Data Availability

The data presented in this study are available on request from the corresponding author.

## Supplementary Materials

The following supporting information can be downloaded at: https://www.mdpi.com/article/10.3390/ph17060810/s1, Figure S1: (**A**) Expression of SIRT3 in MDA-MB-231 scramble, SIRT3-overexpressing (o/e), and SIRT3-silenced (sh) cells. ACTIN is used as a loading control. (**B**) SIRT3 activity in scrambled and SIRT3 clones. (**C**) Expression of SIRT3 in HEK293 scramble, SIRT3-overexpressing, and SIRT3-silenced cells. ACTIN is used as a loading control. (**D**) SIRT3 activity in scrambled and SIRT3 clones. Data are represented as mean ± SD. The bar chart in (**B**,**D**) was compared by the Student’s *t*-test ** *p* < 0.01, and *** *p* < 0.001. Figure S2. (**A**) SIRT3 activity in MDA-MB-231 SIRT3-overexpressing and scrambled mice treated with MC2791. The activity was monitored after 2, 4, 8, and 16 h after the addition of the SIRT3 activator. (**B**) Cell viability of MDA-MB-231 scrambled cells in unbuffered medium at a pH of 7.4 or 6.8 in the presence or absence of SIRT3 activator MC2791. Cell viability was measured for the time indicated. Data are represented as mean ± SD. *, significantly different from time zero, * *p* < 0.05, ** *p* < 0.01. #, significantly different from untreated cells.
