# Supplementary material for: Pharmacological Activation of SIRT3 Modulates the Response of Cancer Cells to Acidic pH
Source: Pharmaceuticals (Basel). 2024 Jun 20;17(6):810. doi: 10.3390/ph17060810 (PMC12365908; doi:10.3390/ph17060810)
Supplement: Supplementary file 1 [file pharmaceuticals-17-00810-s001.zip › pharmaceuticals-3046852-supplementary.pdf]

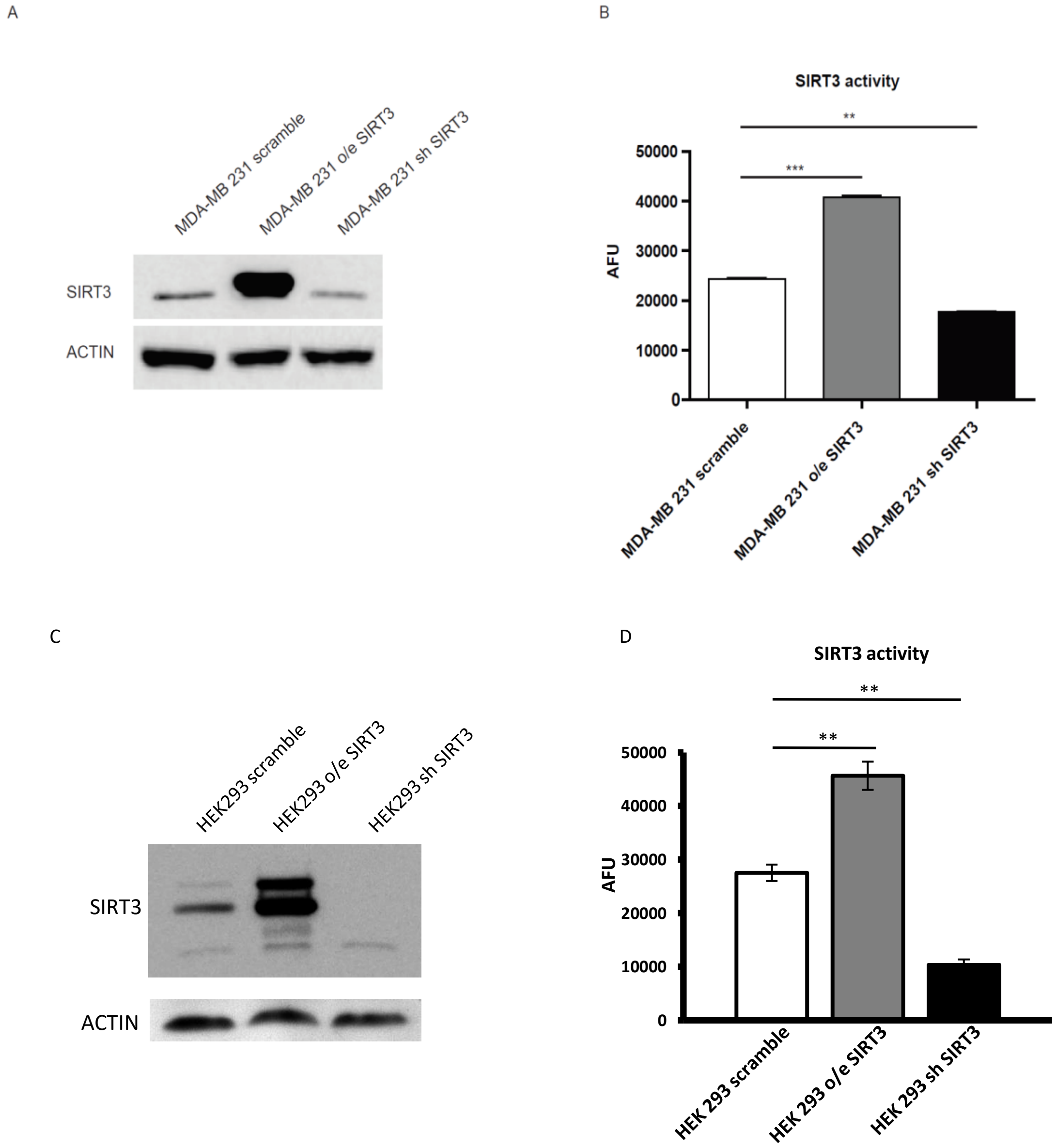

Supplementary Figure S1. (A) Expression of SIRT3 in MDA-MB-231 scramble, SIRT3-overexpressing (o/e) and SIRT3-silenced (sh) cells. ACTIN is used as loading control. (B) SIRT3 activity in scramble and SIRT3 clones. (C) Expression of SIRT3 in HEK293 scramble, SIRT3-overexpressing and SIRT3-silenced cells. ACTIN is used as loading control. (D) SIRT3 activity in scramble and SIRT3 clones. Data are represented as mean ± SEM. The bar chart in (B and D) was compared by Student's t-test $**p < 0.01$, and $***p < 0.001$.

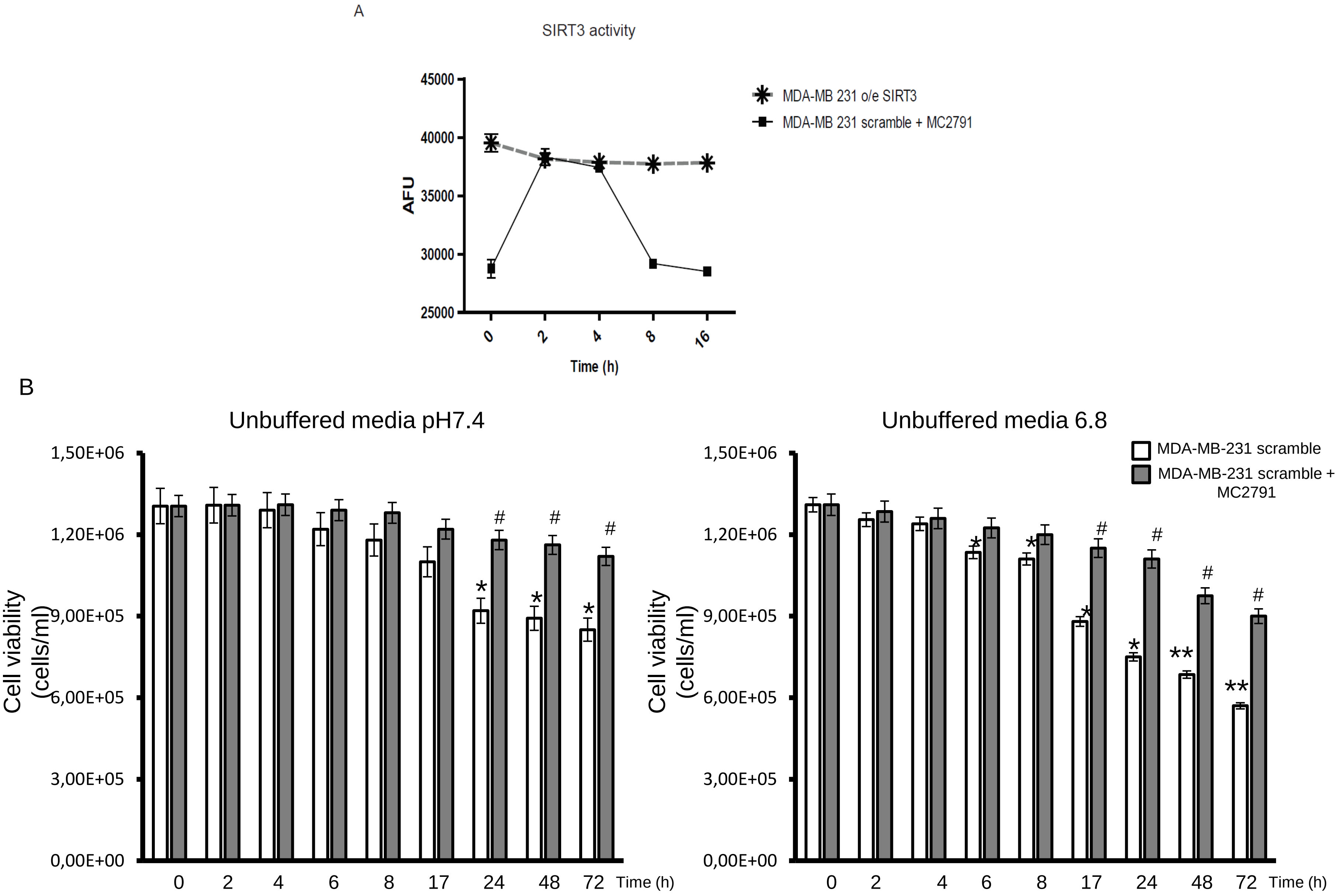

Supplementary Figure S2. (A) SIRT3 activity in MDA-MB-231 SIRT3-overexpressing and scramble treated with MC2791. The activity was monitored after 2, 4, 8 and 16 hours from the addition of SIRT3 activator. (B) Cell viability of MDA-MB-231 scramble cells in unbuffered medium at pH 7.4 or 6.8 in the presence or absence of SIRT3 activator MC2791. Cell viability was measured for the time indicated. Data are represented as mean ± SEM. *, significantly different from time zero, *p<0.05, **p<0.01. #, significantly different from untreated cells.
